# (2*R*)-*N*-(2-Benzoyl­phen­yl)-1-benzyl­pyrrolidine-2-carboxamide

**DOI:** 10.1107/S1600536811033836

**Published:** 2011-08-27

**Authors:** Saira Nayab, Hong-In Lee, Jong Hwa Jeong

**Affiliations:** a Department of Chemistry, Kyungpook National University, Taegu, 702-701, Republic of Korea

## Abstract

In the title compound, C_25_H_24_N_2_O_2_, the dihedral angle between the two benzene rings of the benzophenone moiety is 59.10 (6)°. An intra­molecular, bifurcated N—H⋯(O,N) hydrogen bond, which generates *S*(6) and *S*(5) rings, respectively, helps to establish the overall conformation of the mol­ecule.

## Related literature

For applications of the title compound, see: Deng *et al.* (2008 ▶); Purser *et al.* (2008 ▶). For further synthetic details, see: Tararov *et al.* (1997 ▶); Wang *et al.* (2011 ▶).
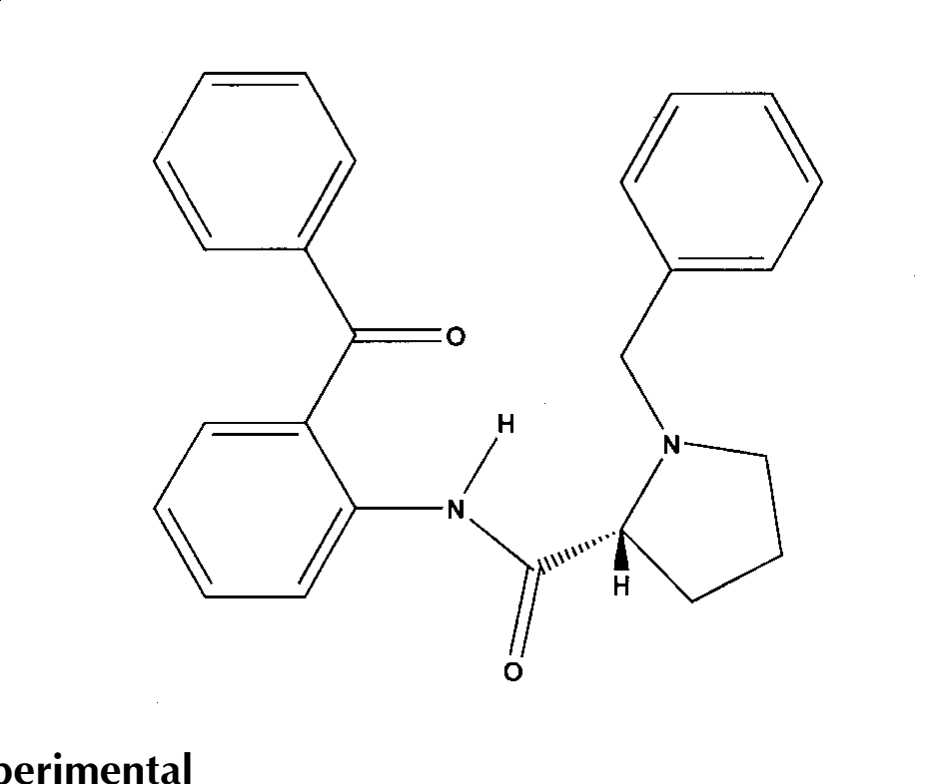


## Experimental

### 

#### Crystal data

C_25_H_24_N_2_O_2_
*M* *_r_* = 384.46
Orthorhombic, 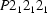

*a* = 8.4036 (7) Å
*b* = 11.2215 (8) Å
*c* = 21.4182 (12) Å
*V* = 2019.8 (2) Å^3^
*Z* = 4
Mo *K*α radiation
μ = 0.08 mm^−1^
*T* = 293 K
0.45 × 0.40 × 0.35 mm

#### Data collection

Enraf–Nonius CAD-4 four-circle diffractometer
4479 measured reflections
3740 independent reflections
2372 reflections with *I* > 2σ(*I*)
*R*_int_ = 0.013
3 standard reflections every 60 min  intensity decay: < 0.2%

#### Refinement

*R*[*F* ^2^ > 2σ(*F* ^2^)] = 0.034
*wR*(*F* ^2^) = 0.084
*S* = 0.93
3740 reflections
266 parameters
H atoms treated by a mixture of independent and constrained refinement
Δρ_max_ = 0.12 e Å^−3^
Δρ_min_ = −0.12 e Å^−3^
Absolute structure: Flack (1983 ▶), 1587 Friedel pairs
Flack parameter: −0.2 (15)

### 

Data collection: *CAD-4 Software* (Enraf–Nonius, 1989 ▶); cell refinement: *CAD-4 Software*; data reduction: *XCAD* (McArdle, 1999 ▶); program(s) used to solve structure: *SHELXS97* (Sheldrick, 2008 ▶); program(s) used to refine structure: *SHELXL97* (Sheldrick, 2008 ▶); molecular graphics: *ORTEPIII* (Burnett & Johnson, 1996 ▶); software used to prepare material for publication: *SHELXL97*.

## Supplementary Material

Crystal structure: contains datablock(s) global, I. DOI: 10.1107/S1600536811033836/hb5950sup1.cif

Structure factors: contains datablock(s) I. DOI: 10.1107/S1600536811033836/hb5950Isup2.hkl

Supplementary material file. DOI: 10.1107/S1600536811033836/hb5950Isup3.cml

Additional supplementary materials:  crystallographic information; 3D view; checkCIF report

## Figures and Tables

**Table 1:** Hydrogen-bond geometry (Å, °)

*D*—H⋯*A*	*D*—H	H⋯*A*	*D*⋯*A*	*D*—H⋯*A*
N2—H2*N*⋯O2	0.855 (19)	2.246 (17)	2.810 (2)	123.5 (15)
N2—H2*N*⋯N1	0.855 (19)	2.209 (18)	2.663 (2)	113.1 (14)

**Fig. 1.**
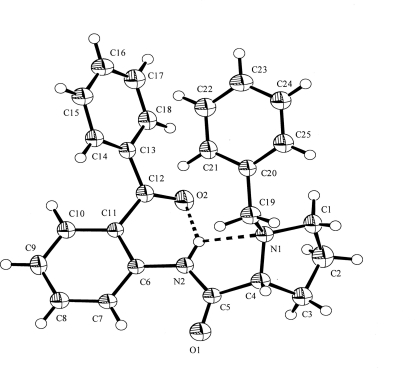
The molecular structure of (I) with displacement ellipsoids drawn at the 40% probability level. Hydrgen bonds are indicated by dashed lines.

## supplementary crystallographic information

### Comment

Asymmetric synthesis of non-proteinogenic amino acids based on the use of chiral auxiliaries has creative potentials in medicinal chemistry (Purser *et al.*, 2008). Among the non-proteinogenic amino acids, 2-[(*N*- benzylpyrrolyl)amino]benzophenone, which can be both simply and stereo- selectively synthesized with low cost, is readily available for the studies of systematic medicinal chemistry (Deng *et al.*, 2008). In this study, we report the crystal structure of the title compound, (I).

Two phenyl groups of benzophenone fragment are rotated from the carbonyl plane due to the steric hindrance caused by the phenyl moiety of the benzyl group. The configuration of C at the pyrrolydine fragment is *R*. There are two intramolecular N-H···O and N-H···N hydrogen bonds forming six and five membered rings, respectively.

### Experimental

The title compound was synthesized by the reported method (Tararov *et al.*, 1997; Wang *et al.*, 2011). Colourless blocks of (I) were obtained through slow diffusion of hexane in to CH_2_Cl_2_ solution at room temperature.

### Refinement

H-atom of N—H was refined isotropically. All H-atoms at C atoms were positioned geometrically and refined using a riding model with *U*_iso_ = 1.2*U*_eq_ (C) for CH_2_, C—H = 0.98 Å, *U*_iso_ = 1.2*U*_eq_ (C) for CH, and C—H = 0.93 Å, *U*_iso_ = 1.2*U*_eq_ (C) for aromatic H-atoms, C—H = 0.93 Å. An absolute structure was tentatively established using anomalous dispersion effects; 1587 Friedel pairs were not merged. Flack *x* parameter for the inverted absolute structure was 1.17.

### Crystal data

**Table:**

C_25_H_24_N_2_O_2_	*F*(000) = 816
*M**_r_* = 384.46	*D*_x_ = 1.264 Mg m^−^^3^
Orthorhombic, *P*2_1_2_1_2_1_	Mo *K*α radiation, λ = 0.71073 Å
Hall symbol: P 2ac 2ab	Cell parameters from 3 reflections
*a* = 8.4036 (7) Å	θ = 9.3–11.9°
*b* = 11.2215 (8) Å	µ = 0.08 mm^−^^1^
*c* = 21.4182 (12) Å	*T* = 293 K
*V* = 2019.8 (2) Å^3^	Block, colorless
*Z* = 4	0.45 × 0.40 × 0.35 mm

### Data collection

**Table:**

Enraf–Nonius CAD-4 four-circle diffractometer	*R*_int_ = 0.013
Radiation source: fine-focus sealed tube	θ_max_ = 25.5°, θ_min_ = 1.9°
graphite	*h* = −10→10
ω/2θ scans	*k* = −13→13
4479 measured reflections	*l* = −25→25
3740 independent reflections	3 standard reflections every 60 min
2372 reflections with *I* > 2σ(*I*)	intensity decay: < 0.2%

### Refinement

**Table:**

Refinement on *F*^2^	Secondary atom site location: difference Fourier map
Least-squares matrix: full	Hydrogen site location: inferred from neighbouring sites
*R*[*F*^2^ > 2σ(*F*^2^)] = 0.034	H atoms treated by a mixture of independent and constrained refinement
*wR*(*F*^2^) = 0.084	*w* = 1/[σ^2^(*F*_o_^2^) + (0.0442*P*)^2^] where *P* = (*F*_o_^2^ + 2*F*_c_^2^)/3
*S* = 0.93	(Δ/σ)_max_ = 0.001
3740 reflections	Δρ_max_ = 0.12 e Å^−^^3^
266 parameters	Δρ_min_ = −0.12 e Å^−^^3^
0 restraints	Absolute structure: Flack (1983), 1587 Friedel pairs
Primary atom site location: structure-invariant direct methods	Flack parameter: −0.2 (15)

### Fractional atomic coordinates and isotropic or equivalent isotropic displacement parameters (Å^2^)

**Table:**

	*x*	*y*	*z*	*U*_iso_*/*U*_eq_	
N1	0.61424 (19)	0.35261 (13)	0.85208 (7)	0.0448 (4)	
O1	0.6320 (2)	0.53634 (14)	0.71393 (7)	0.0686 (5)	
O2	1.0339 (2)	0.48686 (13)	0.88092 (7)	0.0774 (5)	
N2	0.7694 (2)	0.53942 (15)	0.80552 (8)	0.0444 (4)	
H2N	0.792 (2)	0.4945 (17)	0.8365 (9)	0.047 (6)*	
C1	0.6389 (3)	0.22394 (17)	0.85790 (9)	0.0573 (6)	
H1A	0.5382	0.1818	0.8598	0.069*	
H1B	0.7010	0.2050	0.8947	0.069*	
C2	0.7280 (3)	0.1939 (2)	0.79945 (11)	0.0724 (7)	
H2A	0.7201	0.1096	0.7900	0.087*	
H2B	0.8394	0.2156	0.8031	0.087*	
C3	0.6456 (3)	0.26809 (19)	0.75016 (10)	0.0612 (6)	
H3A	0.5577	0.2245	0.7316	0.073*	
H3B	0.7193	0.2910	0.7175	0.073*	
C4	0.5852 (2)	0.37804 (17)	0.78583 (8)	0.0465 (5)	
H4	0.4702	0.3850	0.7793	0.056*	
C5	0.6641 (2)	0.49309 (17)	0.76437 (9)	0.0450 (5)	
C6	0.8458 (2)	0.65120 (16)	0.80547 (8)	0.0382 (4)	
C7	0.8141 (2)	0.73603 (17)	0.76016 (9)	0.0444 (5)	
H7	0.7474	0.7173	0.7269	0.053*	
C8	0.8807 (3)	0.84794 (18)	0.76397 (9)	0.0524 (5)	
H8	0.8594	0.9036	0.7329	0.063*	
C9	0.9778 (3)	0.87866 (17)	0.81265 (10)	0.0542 (5)	
H9	1.0199	0.9551	0.8153	0.065*	
C10	1.0125 (2)	0.79505 (16)	0.85766 (9)	0.0482 (5)	
H10	1.0787	0.8158	0.8907	0.058*	
C11	0.9504 (2)	0.67965 (16)	0.85471 (8)	0.0393 (4)	
C12	1.0078 (2)	0.58769 (18)	0.89961 (9)	0.0468 (5)	
C13	1.0368 (2)	0.61718 (16)	0.96635 (8)	0.0425 (5)	
C14	0.9582 (3)	0.70905 (17)	0.99693 (9)	0.0518 (5)	
H14	0.8901	0.7590	0.9749	0.062*	
C15	0.9812 (3)	0.7261 (2)	1.06040 (9)	0.0650 (6)	
H15	0.9274	0.7868	1.0811	0.078*	
C16	1.0839 (3)	0.6531 (2)	1.09295 (10)	0.0688 (7)	
H16	1.0993	0.6648	1.1355	0.083*	
C17	1.1639 (3)	0.5627 (2)	1.06260 (10)	0.0659 (7)	
H17	1.2347	0.5146	1.0845	0.079*	
C18	1.1388 (3)	0.54412 (18)	1.00017 (9)	0.0537 (5)	
H18	1.1908	0.4817	0.9801	0.064*	
C19	0.4917 (3)	0.40031 (19)	0.89345 (9)	0.0555 (6)	
H19A	0.3930	0.3574	0.8866	0.067*	
H19B	0.4734	0.4835	0.8834	0.067*	
C20	0.5386 (2)	0.38994 (17)	0.96107 (9)	0.0462 (5)	
C21	0.6451 (3)	0.46882 (17)	0.98709 (10)	0.0539 (5)	
H21	0.6849	0.5305	0.9627	0.065*	
C22	0.6942 (3)	0.4592 (2)	1.04770 (10)	0.0637 (6)	
H22	0.7675	0.5130	1.0639	0.076*	
C23	0.6350 (3)	0.3700 (2)	1.08441 (10)	0.0662 (7)	
H23	0.6667	0.3635	1.1259	0.079*	
C24	0.5285 (3)	0.2902 (2)	1.05976 (10)	0.0670 (7)	
H24	0.4892	0.2286	1.0844	0.080*	
C25	0.4798 (3)	0.30099 (18)	0.99869 (10)	0.0596 (6)	
H25	0.4061	0.2473	0.9826	0.071*	

### Atomic displacement parameters (Å^2^)

**Table:**

	*U*^11^	*U*^22^	*U*^33^	*U*^12^	*U*^13^	*U*^23^
N1	0.0480 (9)	0.0427 (9)	0.0435 (8)	0.0001 (8)	0.0027 (8)	0.0000 (7)
O1	0.0901 (12)	0.0611 (9)	0.0546 (9)	−0.0080 (9)	−0.0274 (8)	0.0103 (7)
O2	0.1167 (15)	0.0469 (9)	0.0685 (10)	0.0280 (9)	−0.0326 (10)	−0.0149 (8)
N2	0.0541 (11)	0.0401 (9)	0.0391 (9)	−0.0043 (9)	−0.0060 (8)	0.0050 (8)
C1	0.0671 (15)	0.0447 (12)	0.0601 (13)	−0.0006 (11)	−0.0044 (12)	0.0001 (11)
C2	0.0741 (17)	0.0630 (15)	0.0802 (16)	0.0076 (13)	−0.0003 (14)	−0.0181 (13)
C3	0.0717 (16)	0.0541 (13)	0.0579 (13)	−0.0181 (13)	0.0123 (12)	−0.0147 (11)
C4	0.0442 (12)	0.0509 (12)	0.0445 (10)	−0.0034 (10)	−0.0024 (9)	−0.0037 (9)
C5	0.0472 (12)	0.0463 (11)	0.0414 (10)	0.0041 (10)	−0.0034 (10)	−0.0033 (9)
C6	0.0404 (10)	0.0380 (10)	0.0362 (9)	0.0020 (9)	0.0060 (9)	0.0016 (8)
C7	0.0474 (12)	0.0466 (12)	0.0393 (10)	0.0028 (10)	0.0014 (9)	0.0019 (9)
C8	0.0605 (14)	0.0473 (13)	0.0494 (11)	0.0046 (11)	0.0075 (11)	0.0149 (10)
C9	0.0615 (14)	0.0380 (11)	0.0631 (13)	−0.0092 (11)	0.0109 (12)	0.0033 (10)
C10	0.0514 (13)	0.0435 (11)	0.0495 (11)	−0.0049 (10)	0.0009 (10)	−0.0017 (10)
C11	0.0433 (11)	0.0371 (10)	0.0375 (9)	0.0009 (9)	0.0014 (9)	−0.0007 (8)
C12	0.0484 (13)	0.0415 (11)	0.0505 (11)	0.0008 (10)	−0.0063 (10)	−0.0021 (9)
C13	0.0431 (11)	0.0372 (10)	0.0474 (10)	−0.0079 (10)	−0.0071 (9)	0.0038 (9)
C14	0.0594 (14)	0.0453 (11)	0.0508 (11)	−0.0005 (11)	−0.0034 (11)	0.0011 (10)
C15	0.0819 (17)	0.0612 (14)	0.0519 (13)	−0.0120 (14)	0.0030 (12)	−0.0092 (11)
C16	0.0921 (19)	0.0694 (16)	0.0448 (11)	−0.0304 (16)	−0.0156 (12)	0.0042 (12)
C17	0.0756 (16)	0.0594 (15)	0.0627 (15)	−0.0169 (14)	−0.0253 (13)	0.0166 (12)
C18	0.0542 (13)	0.0455 (10)	0.0615 (13)	−0.0057 (11)	−0.0164 (11)	0.0050 (10)
C19	0.0592 (15)	0.0551 (13)	0.0521 (12)	0.0053 (12)	0.0052 (11)	0.0012 (10)
C20	0.0483 (12)	0.0414 (12)	0.0489 (11)	0.0009 (11)	0.0075 (10)	−0.0020 (9)
C21	0.0583 (14)	0.0427 (11)	0.0608 (13)	−0.0006 (11)	0.0109 (11)	−0.0001 (10)
C22	0.0692 (16)	0.0613 (14)	0.0605 (14)	0.0017 (13)	−0.0013 (12)	−0.0158 (13)
C23	0.0763 (17)	0.0746 (16)	0.0479 (12)	0.0274 (15)	−0.0007 (12)	−0.0090 (12)
C24	0.0869 (18)	0.0568 (14)	0.0573 (14)	0.0066 (14)	0.0209 (13)	0.0180 (11)
C25	0.0643 (15)	0.0546 (12)	0.0598 (13)	−0.0105 (12)	0.0098 (12)	0.0000 (11)

### Geometric parameters (Å, °)

**Table:**

N1—C19	1.460 (2)	C10—H10	0.9300
N1—C1	1.464 (2)	C11—C12	1.491 (3)
N1—C4	1.468 (2)	C12—C13	1.487 (3)
O1—C5	1.215 (2)	C13—C14	1.389 (3)
O2—C12	1.220 (2)	C13—C18	1.390 (3)
N2—C5	1.353 (2)	C14—C15	1.386 (3)
N2—C6	1.409 (2)	C14—H14	0.9300
N2—H2N	0.855 (19)	C15—C16	1.379 (3)
C1—C2	1.497 (3)	C15—H15	0.9300
C1—H1A	0.9700	C16—C17	1.379 (3)
C1—H1B	0.9700	C16—H16	0.9300
C2—C3	1.512 (3)	C17—C18	1.370 (3)
C2—H2A	0.9700	C17—H17	0.9300
C2—H2B	0.9700	C18—H18	0.9300
C3—C4	1.537 (3)	C19—C20	1.505 (3)
C3—H3A	0.9700	C19—H19A	0.9700
C3—H3B	0.9700	C19—H19B	0.9700
C4—C5	1.522 (3)	C20—C25	1.374 (3)
C4—H4	0.9800	C20—C21	1.377 (3)
C6—C7	1.385 (2)	C21—C22	1.366 (3)
C6—C11	1.410 (2)	C21—H21	0.9300
C7—C8	1.377 (3)	C22—C23	1.366 (3)
C7—H7	0.9300	C22—H22	0.9300
C8—C9	1.368 (3)	C23—C24	1.372 (3)
C8—H8	0.9300	C23—H23	0.9300
C9—C10	1.376 (3)	C24—C25	1.376 (3)
C9—H9	0.9300	C24—H24	0.9300
C10—C11	1.397 (3)	C25—H25	0.9300
			
C19—N1—C1	114.18 (16)	C10—C11—C6	118.45 (16)
C19—N1—C4	113.48 (15)	C10—C11—C12	119.44 (17)
C1—N1—C4	107.31 (14)	C6—C11—C12	121.85 (17)
C5—N2—C6	129.77 (17)	O2—C12—C13	119.48 (18)
C5—N2—H2N	115.1 (13)	O2—C12—C11	119.24 (17)
C6—N2—H2N	115.2 (13)	C13—C12—C11	121.26 (17)
N1—C1—C2	102.79 (17)	C14—C13—C18	119.06 (18)
N1—C1—H1A	111.2	C14—C13—C12	122.72 (18)
C2—C1—H1A	111.2	C18—C13—C12	118.07 (17)
N1—C1—H1B	111.2	C15—C14—C13	119.9 (2)
C2—C1—H1B	111.2	C15—C14—H14	120.1
H1A—C1—H1B	109.1	C13—C14—H14	120.1
C1—C2—C3	103.34 (19)	C16—C15—C14	120.1 (2)
C1—C2—H2A	111.1	C16—C15—H15	120.0
C3—C2—H2A	111.1	C14—C15—H15	120.0
C1—C2—H2B	111.1	C17—C16—C15	120.2 (2)
C3—C2—H2B	111.1	C17—C16—H16	119.9
H2A—C2—H2B	109.1	C15—C16—H16	119.9
C2—C3—C4	104.22 (17)	C18—C17—C16	119.8 (2)
C2—C3—H3A	110.9	C18—C17—H17	120.1
C4—C3—H3A	110.9	C16—C17—H17	120.1
C2—C3—H3B	110.9	C17—C18—C13	120.9 (2)
C4—C3—H3B	110.9	C17—C18—H18	119.5
H3A—C3—H3B	108.9	C13—C18—H18	119.5
N1—C4—C5	112.61 (16)	N1—C19—C20	111.80 (17)
N1—C4—C3	105.65 (15)	N1—C19—H19A	109.3
C5—C4—C3	112.77 (15)	C20—C19—H19A	109.3
N1—C4—H4	108.6	N1—C19—H19B	109.3
C5—C4—H4	108.6	C20—C19—H19B	109.3
C3—C4—H4	108.6	H19A—C19—H19B	107.9
O1—C5—N2	124.84 (19)	C25—C20—C21	117.60 (19)
O1—C5—C4	120.71 (18)	C25—C20—C19	121.75 (19)
N2—C5—C4	114.45 (17)	C21—C20—C19	120.64 (19)
C7—C6—N2	121.63 (17)	C22—C21—C20	122.0 (2)
C7—C6—C11	119.24 (17)	C22—C21—H21	119.0
N2—C6—C11	119.03 (16)	C20—C21—H21	119.0
C8—C7—C6	120.45 (19)	C23—C22—C21	119.7 (2)
C8—C7—H7	119.8	C23—C22—H22	120.2
C6—C7—H7	119.8	C21—C22—H22	120.2
C9—C8—C7	121.15 (18)	C22—C23—C24	119.6 (2)
C9—C8—H8	119.4	C22—C23—H23	120.2
C7—C8—H8	119.4	C24—C23—H23	120.2
C8—C9—C10	119.23 (18)	C23—C24—C25	120.1 (2)
C8—C9—H9	120.4	C23—C24—H24	119.9
C10—C9—H9	120.4	C25—C24—H24	119.9
C9—C10—C11	121.39 (18)	C20—C25—C24	121.0 (2)
C9—C10—H10	119.3	C20—C25—H25	119.5
C11—C10—H10	119.3	C24—C25—H25	119.5
			
C19—N1—C1—C2	163.29 (17)	C10—C11—C12—O2	−138.3 (2)
C4—N1—C1—C2	36.6 (2)	C6—C11—C12—O2	35.8 (3)
N1—C1—C2—C3	−40.8 (2)	C10—C11—C12—C13	40.5 (3)
C1—C2—C3—C4	29.9 (2)	C6—C11—C12—C13	−145.40 (19)
C19—N1—C4—C5	91.8 (2)	O2—C12—C13—C14	−154.8 (2)
C1—N1—C4—C5	−141.14 (18)	C11—C12—C13—C14	26.4 (3)
C19—N1—C4—C3	−144.72 (17)	O2—C12—C13—C18	20.7 (3)
C1—N1—C4—C3	−17.6 (2)	C11—C12—C13—C18	−158.10 (19)
C2—C3—C4—N1	−8.0 (2)	C18—C13—C14—C15	−0.5 (3)
C2—C3—C4—C5	115.4 (2)	C12—C13—C14—C15	174.96 (19)
C6—N2—C5—O1	10.4 (3)	C13—C14—C15—C16	1.0 (3)
C6—N2—C5—C4	−169.86 (17)	C14—C15—C16—C17	−0.1 (3)
N1—C4—C5—O1	−168.60 (18)	C15—C16—C17—C18	−1.3 (3)
C3—C4—C5—O1	71.9 (2)	C16—C17—C18—C13	1.8 (3)
N1—C4—C5—N2	11.6 (2)	C14—C13—C18—C17	−0.9 (3)
C3—C4—C5—N2	−107.8 (2)	C12—C13—C18—C17	−176.5 (2)
C5—N2—C6—C7	2.7 (3)	C1—N1—C19—C20	65.8 (2)
C5—N2—C6—C11	179.17 (19)	C4—N1—C19—C20	−170.79 (16)
N2—C6—C7—C8	174.78 (17)	N1—C19—C20—C25	−100.8 (2)
C11—C6—C7—C8	−1.7 (3)	N1—C19—C20—C21	78.0 (2)
C6—C7—C8—C9	−0.8 (3)	C25—C20—C21—C22	1.1 (3)
C7—C8—C9—C10	1.7 (3)	C19—C20—C21—C22	−177.7 (2)
C8—C9—C10—C11	−0.1 (3)	C20—C21—C22—C23	−1.0 (3)
C9—C10—C11—C6	−2.3 (3)	C21—C22—C23—C24	0.9 (3)
C9—C10—C11—C12	172.00 (18)	C22—C23—C24—C25	−1.0 (3)
C7—C6—C11—C10	3.1 (3)	C21—C20—C25—C24	−1.3 (3)
N2—C6—C11—C10	−173.41 (17)	C19—C20—C25—C24	177.5 (2)
C7—C6—C11—C12	−170.99 (17)	C23—C24—C25—C20	1.2 (3)
N2—C6—C11—C12	12.5 (3)		

### Hydrogen-bond geometry (Å, °)

**Table:**

*D*—H···*A*	*D*—H	H···*A*	*D*···*A*	*D*—H···*A*
N2—H2N···O2	0.855 (19)	2.246 (17)	2.810 (2)	123.5 (15)
N2—H2N···N1	0.855 (19)	2.209 (18)	2.663 (2)	113.1 (14)

## References

[bb1] Burnett, M. N. & Johnson, C. K. (1996). *ORTEPIII.* Report ORNL-6895. Oak Ridge National Laboratory, Tennessee, USA.

[bb2] Deng, G., Ye, D., Li, Y., He, L., Zhou, Y., Wang, J., Li, J., Jiang, H. & Liu, H. (2008). *Tetrahedron*, **64**, 10512–10516.

[bb3] Enraf–Nonius (1989). *CAD-4 Software.* Version 5.0. Enraf–Nonius, Delft, The Netherlands.

[bb4] Flack, H. D. (1983). *Acta Cryst.* A**39**, 876–881.

[bb5] McArdle, P. (1999). *XCAD* National University of Ireland, Galway, Ireland.

[bb6] Purser, S., Moore, P. R., Swallow, S. & Gouverneur, V. (2008). *Chem. Soc. Rev.* **37**, 320–330. 10.1039/b610213c 18197348

[bb7] Sheldrick, G. M. (2008). *Acta Cryst.* A**64**, 112–122. 10.1107/S0108767307043930 18156677

[bb8] Tararov, V. I., Saveleva, T. F., Kuznetsov, N. Y., Ikonnikov, N. S., Orlova, S. A., Belokon, Y. N. & North, M. (1997). *Tetrahedron Asymmetry*, **8**, 79–83.

[bb9] Wang, J., Lin, D., Zhou, S., Ding, X., Soloshonok, V. A. & Liu, H. (2011). *J. Org. Chem.* **76**, 684–687. 10.1021/jo102031b 21182272

